# Subinhibitory Concentrations of Fusidic Acid May Reduce the Virulence of *S. aureus* by Down-Regulating *sarA* and *saeRS* to Reduce Biofilm Formation and α-Toxin Expression

**DOI:** 10.3389/fmicb.2020.00025

**Published:** 2020-02-14

**Authors:** Li Liu, Xiaofei Shen, Jingyi Yu, Xingwei Cao, Qing Zhan, Yinjuan Guo, Fangyou Yu

**Affiliations:** ^1^Department of Laboratory Medicine, The First Affiliated Hospital of Wenzhou Medical University, Wenzhou, China; ^2^Department of Respiratory Medicine, The First Affiliated Hospital of Wenzhou Medical University, Wenzhou, China; ^3^Jiangxi Provincial Key Laboratory of Medicine, Clinical Laboratory of the Second Affiliated Hospital of Nanchang University, Nanchang, China; ^4^Jiangxi Provincial Key Laboratory of Preventive Medicine, Nanchang University, Nanchang, China; ^5^Department of Clinical Laboratory, Shanghai Pulmonary Hospital, Tongji University School of Medicine, Shanghai, China; ^6^Shanghai Key Laboratory of Tuberculosis, Shanghai Pulmonary Hospital, Tongji University School of Medicine, Shanghai, China

**Keywords:** subinhibitory concentration fusidic acid, biofilm, *saeRS*, *sarA*, α-toxin

## Abstract

*Staphylococcus aureus* is an important pathogen in hospital and community infections. Fusidic acid is particularly effective in treating skin and wound infections caused by staphylococci. The purpose of our study was to clarify the effect of fusidic acid on the biofilm formation and α-toxin expression of *S. aureus* at subinhibitory concentrations [1/64, 1/32, and 1/16 × minimum inhibitory concentration (MIC)]. A total of 504 genes greater than a twofold or less than twofold change in expression of *S. aureus* effected by subinhibitory concentrations of fusidic acid were found, including 232 up-regulated genes and 272 down-regulated genes, which were determined by transcriptome sequencing. Our results showed subinhibitory concentrations of fusidic acid significantly inhibited the expression of *hla*, *spa*, *icaA*, and *cidA* at the mRNA level in clinical *S. aureus* strains tested. And subinhibitory concentrations of fusidic acid can significantly reduce the hemolysis activity and α-toxin production of *S. aureus*. In addition, the subinhibitory concentrations of fusidic acid significantly inhibited biofilm formation, autolysis, cell aggregation, and polysaccharide intercellular adhesin (PIA) production of *S. aureus*. Moreover, fusidic acid effectively reduces the damage of mouse skin lesion area. Furthermore, fusidic acid reduced the expression of the two-component regulatory system *saeRS* and staphylococcal accessory gene regulator (*sarA*). In conclusion, our results suggested that the subinhibitory concentrations of fusidic acid may reduce the virulence of *S. aureus* by down-regulating *sarA* and *saeRS* to reduce biofilm formation and α-toxin expression, which will provide a theoretical basis for the clinical treatment of *S. aureus* infection. This is the first report that fusidic acid has an inhibitory effect on the virulence of *S. aureus*, and this broadens the clinical application of fusidic acid.

## Introduction

*Staphylococcus aureus* is an important pathogen of humans, causing infections ranging from small skin infections to osteomyelitis, sepsis, and necrotizing pneumonia (Lowy, 1998). With the emergence of methicillin-resistant *S. aureus* (MRSA), the treatment of *S. aureus* infection has become one of the clinically difficult problems, and it is increasingly threatening the health of the public (Okesola, 2011). It is well-known that the production of toxins and the formation of biofilms are two important contributions to *S. aureus* infection. Nowadays, there is a strategy for treating *S. aureus* infections that reduces the virulence of *S. aureus* rather than the direct killing of *S. aureus* in the past (Clatworthy et al., 2007; Rasko and Sperandio, 2010). However, whether the use of subinhibitory antibiotics can effectively reduce the resistance rate of *S. aureus* is still controversial (Bhattacharya et al., 2017), and further research is needed. *S. aureus* produces a variety of toxins (Duan et al., 2018), wherein α-toxin acts as an indispensable part of the pathogenicity of *S. aureus* leading to tissue damage. The α-toxin is 33.2 kDa of exocrine protein encoded by *hla* gene, which is also a pore-forming toxin that causes cell damage and death (Bhakdi and Tranum-Jensen, 1991). It has obvious hemolysis effects on red blood cells of various mammals, especially on rabbit red blood cells (Kebaier et al., 2012). Biofilm is a community of microbial cells that are usually attached to a surface and joined together by an outer cavity matrix (DeFrancesco et al., 2017). Biofilms formed by pathogens, including staphylococci, have been shown to contribute to the refractory nature of infection, in part because of the increased tolerance of antibiotics conferred by the matrix itself, which reduces the penetration of antimicrobial agents (Conlon, 2014; Stewart, 2015). The way antibiotics alter the production of biofilms is usually by altering the expression of genes involved in biofilm formation (Hodille et al., 2017). For example, there are reports in the literature that the expression of major adhesion genes is up-regulated after *S. aureus* exposure to 1/4 × MIC clindamycin-induced biofilm (Schilcher et al., 2016). The current literature on subinhibitory concentrations of antibiotics to reduce biofilm formation is relatively rare. Therefore, if antibiotics can be used to reduce the production of *S. aureus* virulence factors and biofilm formation, it will greatly benefit the treatment of *S. aureus* infection.

Fusidic acid is an antibiotic produced by the *Fusidium coccineum* fungus, which is characterized by an α,β-unsaturated carboxylic acid; its molecular formula is C_31_H_48_O_6_, containing an acetoxy group and two hydroxyl groups (Godtfredsen et al., 1962), belonging to steroids, but without corticosteroids. *In vitro* resistance to fusidic acid was first observed in Guenthner and Wenzel (1984); although the drug was widely used, it remained at an extremely low level (Chen et al., 2010). Nowadays, it is used for the treatment of infections caused by MRSA. In addition, it is a hypoallergenic drug with low toxicity, low drug resistance, and no cross-resistance with other clinically used antibiotics (Curbete and Salgado, 2016).

In the present study, we selected three fusidic acid-resistant clinical *S. aureus* isolates named JP21, JP35, and JP45 to investigate the effect of subinhibitory concentrations of fusidic acid on the virulence of *S. aureus*. The purpose of this experiment was to elucidate that the subinhibitory concentrations of fusidic acid can inhibit the production α-toxin and biofilm formation of *S. aureus.*

## Materials and Methods

### Bacterial Strains and Reagents

The strains used in the experiments were three clinical *Staphylococcus aureus* isolates named JP21 belonging to ST7, JP35 belonging to ST641, and JP45 belonging to ST59, with high-level fusidic acid resistance. JP35 and JP45 belong to MRSA, whereas JP21 belongs to MSSA. The reason why JP21, JP45, and JP35 are resistant to fusidic acid is the mutation of *fusA* gene. On the basis of their production of α-toxin and biofilm formation, we used JP21 and JP45 to study the effects of fusidic acid on α-toxin and JP21 and JP35 for biofilm research. Fusidic acid was purchased from Solarbio (BJ, China). The fusidic acid solution used in the experiments was dissolved in 95% ethanol. Strains were primarily isolated as part of a previous study. All strains were isolated from The First Affiliated Hospital of Wenzhou Medical University. The medical records of patients and *S. aureus* isolates were obtained for research purposes and approved by the Ethics Committee of The First Affiliated Hospital of Wenzhou Medical University. And written informed consent was obtained from patients.

### Determination of the Minimum Inhibitory Concentration of Fusidic Acid

The fusidic acid used in our experiments was first dissolved in 95% ethanol at 5 mg/ml, and the minimum inhibitory concentration (MIC) was determined by the method of CLSI guidelines (CLSI, 2018). In order to rule out the effect of the solvent on the assay, we tested the same amount of solvent as a control.

### Growth Assay

The *S. aureus* strains were first activated twice and added to 200 ml of tryptic soy broth (TSB) (BD, NJ, United States) at a ratio of 1:200 and cultured to an optical density (OD) value of 0.3 at 600 nm. The culture was then divided into four Erlenmeyer flasks, and different amounts of fusidic acid were added to give a final concentration of 1/64, 1/32, and 1/16 × MIC (8, 16, and 32 μg/ml for JP21 and JP35; and 4, 8, and 16 μg/ml for JP45). In addition, a growth curve is prepared with the same amount of drug solvent (95% ethanol) as the drug to verify whether 95% ethanol affects bacterial growth. A conical flask with only TSB was used as a blank control. All cultures were incubated at 37°C with shaking at 220 r.p.m. and the OD_600_ values were measured every hour for a total of 24 h. The assay was performed in triplicate.

### RNA-seq and Identification of Differentially Expressed Genes

Bacteria were cultured for 16 h in TSB supplemented with 1/32 × MIC of fusidic acid or without fusidic acid and then were collected by centrifugation at 12,000 *g* for 1 min at 4°C. RNA was then extracted according to the method recommended by the manufacturer of the kit QIAGEN RNeasy Maxi column (QIAGEN, Berlin, Germany). The tested RNA was analyzed by RNA sequencing using Illumina HiSeq X platform and pe150 (150-bp double-stranded assay) strategy, and DEGseq software was used to analyze the effect of fusidic acid on gene expression. Usually we think | log2 (folding change)| > 1, and *p* < 0.005 represents the difference in gene expression between samples.

### Quantitative Enzyme-Linked Immunosorbent Assay for α-Toxin

The bacteria were cultured in TSB to an OD_600_ of 0.3, and then various concentrations of fusidic acid were added and cultured for 24 h. Thereafter, the supernatant was collected by centrifugation for 2 min at 6,000 *g* and filtered with a 0.22-μm filter for use. We used a staphylococcal α-toxin Elisa kit (Sigma-Aldrich, St. Louis, MO, United States) to detect α-toxin. The above extracted supernatant was added to the well, and then a horseradish peroxidase (HRP) marker was added to the above plate, and these mixtures became an antibody–antigenase-labeled antibody complex. After being washed, a TMB (3,3′,5,5′-tetramethylbenzidine) substrate solution was added, the reaction was terminated by the addition of a sulfuric acid solution, and the color change was measured spectrophotometrically at a wavelength of 450 nm. The concentration of α-toxin in each sample was calculated using the standard curve *y* = *ax* + *b* and then multiplied by the corresponding dilution factor. Each test was performed in triplicate.

### α-Toxin Activity Determination

α-Toxin activity determination was used to determine the inhibitory effect of fusidic acid on the release of α-toxin. The experimental method is based on the previous literature and has been slightly modified (Teng et al., 2017). *S. aureus* strains were cultured at 37°C in TSB containing different concentrations of fusidic acid and no fusidic acid. And the supernatant was extracted according to the above experimental method. Then 100 μl of supernatant was added to 875 μl of hemolysis buffer, incubated with 25 μl of defibrinated rabbit blood for 1 h at 37°C, and centrifuged at 6,000 *g* for 2 min; the supernatant was taken; and the OD value of the supernatants was measured at 600 nm. Triton X-100 was used as a positive control, and 0.9% NaCl was used as a negative control. In order to rule out the effect of the solvent on the hemolytic activity of *S. aureus*, we added the same dose of solvent as the drug to observe whether the hemolytic activity was changed. Each test was performed independently in triplicate.

### Real-Time Polymerase Chain Reaction

*Staphylococcus aureus* strains were cultured in TSB supplemented with different concentrations of fusidic acid. After 16 h, RNA was extracted according to the method described above. The primer pairs used in real-time RT-PCR are listed in Supplementary Table S1. The cDNA was synthesized from total RNA using a Takara RNA PCR kit (Takara, Tokyo, Japan). PCRs were performed in 20 μl of reaction mixtures using Luna Universal qPCR Master Mix (NEB, MA, United States). Each test was performed independently in triplicate.

### Biofilm Semi-Quantitative Assay

The method refers to the previous literature and makes minor adjustments (Tremblay et al., 2013). Strains were cultured in TSB at 37°C with shaking at 220 r.p.m., and then a ratio of 1:100 was added to 200 μl of TSB, and different doses of fusidic acid were added to the medium to obtain a final concentration of 1/64, 1/32, and 1/16 × MIC. The test was carried out in a 96-well plate with three attachment holes per well. In a 37°C incubator, the cultures were allowed to stand for 24 h, and the OD value was measured at OD_600_ and make sure the amount of bacteria is the same before proceeding to the next step. Then the supernatant was discarded and washed three times with sterile phosphate-buffered saline (PBS), and then 99% methanol was added for 15 min; the supernatant was discarded, and 2% crystal violet was added for 10 min; the stained holes were gently rinsed with tap water until the water is colorless. After being dried, 70% glacial acetic acid was added, and the OD value was measured at OD_490_. *Staphylococcus epidermidis* 12228 and 35984 were used as a negative control and a positive control, respectively, and TSB was the blank control. Prior to this, the effect of 1/512 to 1/4 × MIC concentration of fusidic acid on JP21 biofilm was examined by the same method. In order to eliminate the effect of solvents on the biofilm formation of *S. aureus*, we added the same dose of solvent as the drug to observe whether the biofilm formation changed. The assay was performed in triplicate.

### Scanning Electron Microscopy

A single colony of *S. aureus* strain was picked, inoculated in 5 ml of TSB medium at 37°C with shaking at 220 r.p.m. overnight, and then placed in 20 ml of TSB at a ratio of 1:200. Four sterile coverslips were placed in a six-well cell culture plate, 3 ml of the diluted bacterial solution was added, and the corresponding amount of fusidic acid was added to the next three wells to a final concentration of 1/16, 1/32, and 1/64 × MIC, with static culture at 37°C for 24 h. The bacteria were cultured under the same conditions for colony counting to ensure that the amount of bacteria was consistent and the next experiment was carried out. The bacterial solution was drained, the coverslip was taken out, and the unadhered bacteria were removed by washing three times with a sterile PBS, fixed in a 2.5% glutaraldehyde fixative for 2 h, and washed again three times with sterile PBS. They were then dehydrated with 50, 70, 80, 90, and 10% absolute ethanol for 15 min. The coverslips were placed in a sample box and placed in a freeze-drying apparatus for 2 h, and the gold spray was taken out for 3 min, and observed by scanning electron microscopy.

### Triton X-100-Induced Autolysis Assay

*Staphylococcus aureus* strains were cultured in TSB overnight, then a ratio of 1:200 was added to 200 ml of TSB containing 1 M of NaCl; when the OD value of the cultures reached up to 0.3 at 600 nm, the cultures were aliquoted into four flasks. Varying doses of fusidic acid were added to three of the cultures to obtain final concentrations of 1/64, 1/32, and 1/16 × MIC. The culture was continuously to shaken until the OD value was 0.7–0.8 at 600 nm; the bacteria were collected by centrifugation at 4°C for 15 min at 6,000 *g*. The bacteria were resuspended in 15 ml of deionized water and centrifuged, and the process was repeated three times. After the supernatant was discarded for the last time, it was resuspended in a buffer containing 0.05% Triton X-100 (50 mM Tris-Cl, pH = 7.2), and the OD_600_ value was adjusted to 1.0, followed by shaking of the culture at 30°C, 220 r.p.m. The OD_600_ value was measured every 0.5 h with the *S. epidermidis* ΔatlE mutant as a control and continuously monitored for 3 h. The assay was performed in triplicate.

### Cell Aggregation Assay

Cell aggregation was analyzed as previously reported (Feldman et al., 2018). Three strains of *S. aureus* were added to 2 ml of TSB at a ratio of 1:200; then the corresponding fusidic acid was added to give a final concentration of 1/64, 1/32, and 1/16 × MIC; and the culture was shaken at 250 r.p.m. and cultivated for 24 h. Untreated samples were used as controls. The bacteria were then collected by centrifugation at 16,600 *g* for 2 min, and the bacteria were washed three times with PBS. The washed bacteria were resuspended in 3 ml of PBS, and the OD value at 595 nm = 1.5 (OD initial value) was adjusted in a clean glass tube and allowed to stand at room temperature for 24 h. Next, the supernatant was gently aspirated, and the aggregated pellet was resuspended in 3 ml of PBS. The turbidity of the aggregates was measured at OD_595_ (OD final value) using a microplate reader. The aggregation percentage is determined as OD final/OD initial × 100%. The relative aggregation of the treated samples is expressed as a percentage of the untreated control (100%). The assay was performed in triplicate.

### Enzyme-Linked Dot Immunoblot Assay for Polysaccharide Intercellular Adhesin

The experimental method refers to the previous literature and slightly adjusts accordingly (Schlag et al., 2007). In short, 2 ml of TSB was added to a six-well plate; the overnight shocked cultured *S. aureus* was diluted to 10^7^ colony-forming unit (CFU) and added to the above 2 ml of TSB at a ratio of 1:100; and the corresponding fusidic acid was added to make the final concentration at 1/8, 1/16, and 1/32 × MIC. The culture was allowed to stand at 37°C for 24 h. The medium was then aspirated, and the bottom biofilm was suspended by the addition of 1 ml of EDTA. It was transferred to a 1.5-ml EP tube and cooked for 5 min in a 100°C water bath. The supernatant was then centrifuged at 6,000 *g* for 1 min, and 10 μl of 20 mg/ml proteinase K was added per 40 μl of the supernatant, and a 37°C water bath was used for 2 h. A suitable size of polyvinylidene difluoride (PVDF) membrane was cut, soaked in formaldehyde for 3 min, and then soaked in deionized water for 15 min to activate the group on the PVDF membrane. Ten microliters of the extracted polysaccharide intercellular adhesin (PIA) sample was pipetted onto the treated PVDF membrane, thoroughly dried, and transferred to a blocking solution [3.5% bovine serum albumin (BSA) in PBST], and the membrane was completely immersed in the blocking solution at 4°C overnight. The next day, the membrane was taken out, placed in a dish containing wheat germ agglutinin conjugated to HRP (WGA-HRP), and incubated at 37°C for 1 h. The membrane was then removed by rinsing twice with PBST for 12 min per rinse and then once with PBS. Then color developed with enhanced chemiluminescence (ECL) luminescent liquid.

### Mouse Skin Abscess Model

Animal studies were approved by the Institutional Animal Care and Use Committee. The ethical review was passed by the Ethics Committee of Wenzhou Medical University. Male 4- to 6-week-old BALB/c mice were used in the study, with four mice in each group. The specific method refers to the previously published article (Shang et al., 2019). BALB/c mice were completely anesthetized with 1% (mass/volume) sodium pentobarbital (50 mg/kg body weight), and the hair on their back was removed with a depilatory cream. Then, 100 μl of 5 × 10^8^ CFU JP21 was inoculated subcutaneously in the back of the mouse and then randomly divided into two groups. The mice in the treatment group were intraperitoneally injected with 1 μg of fusidic acid per gram of body weight once a day for 14 days. Mice injected with PBS served as a blank control. The area assessed by the maximum length and width of the developing ulcer was measured daily. The lesion area was calculated using the formula *A* = *L* × *W*, where *L* is the length and *W* is the width.

### Statistical Analysis

Experimental data were analyzed using GraphPad Prism6 software (version 6.00, La Jolla, CA, United States). A *p*-value less than 0.05 was considered to be statistically significant. In addition to the growth curve and the autolysis curve using multiple *t*-tests, the others used one-way ANOVA.

## Results

### Influence of Subinhibitory Concentrations of Fusidic Acid on the Growth of *Staphylococcus aureus* Strains

**FIGURE 1:**
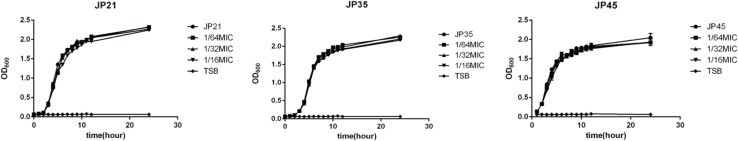
Growth curves of *Staphylococcus aureus* strains cultured with different subinhibitory concentrations of fusidic acid (1/64, 1/32, and 1/16 × MIC), with tryptic soy broth (TSB) as a blank control.

The MIC values of fusidic acid against *Staphylococcus aureus* JP21, JP35, and JP45 were 521, 512, and 256 μg/ml, respectively. Higher subinhibitory concentrations of fusidic acid inhibited the growth of *S. aureus* clinical isolates tested (Supplementary Figure S1). To ensure that fusidic acid reduces the virulence of *S. aureus* by reducing the expression of virulence genes rather than reducing the amount of bacteria, we chose these three concentrations (1/16, 1/32, and 1/64 × MIC), which did not affect the growth of *S. aureus* isolates in follow-up studies. At the subinhibitory concentrations (1/16, 1/32, and 1/64 × MIC) of fusidic acid, the amount of bacteria at the late logarithmic growth phase was consistent (Figure 1).

### Regulation of a Subinhibitory Concentration of Fusidic Acid on the Main Important Genes of *Staphylococcus aureus*

**FIGURE 2:**
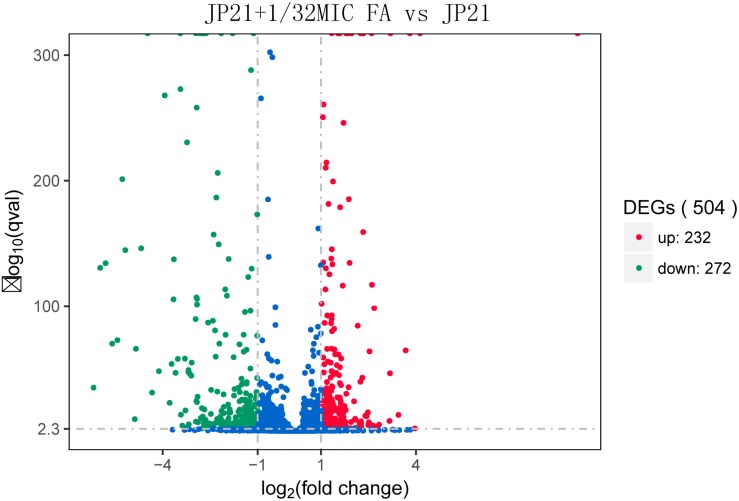
Volcano plot of differences in gene expression between JP21 and JP21 + 1/32 × minimum inhibitory concentration (MIC) FA. The abscissa refers to the fold-change in the two samples; the ordinate refers to the statistically significant difference in gene expression; red dots indicate a significant difference in up-regulated genes; and green dots indicate down-regulated genes.

After determining the MIC value, we selected *S. aureus* JP21 treated with 1/32 × MIC fusidic acid for transcriptome sequencing analysis. RNA samples extracted from JP21 treated with fusidic acid (JP21 added with 1/32 × MIC fusidic acid) and fusidic acid-untreated JP21 were sequenced on an Illumina HiSeq X platform using a pe150 strategy. According to the standard criteria of | log2 (fold change)| > 1 and *q* value < 0.005, 504 genes were found to have differential expression (expression difference greater than a twofold change), including 232 up-regulated genes and 272 down-regulated genes (Figure 2). In addition, fusidic acid down-regulated the expression of genes involved in virulence, two-component regulatory systems, and transcriptional regulation (Table 1).

**TABLE 1:** The expression changes of several important genes associated with two-component systems, transcription regulator, virulence, and cell adhesion, after treatment with fusidic acid.

**Gene_id**	**log2(Fold_change)**	***p*-value**	***q* value**	**Description**
**Two-component system**				
SAOUHSC_02098	1.8525	9.03E-37	4.06E-36	DNA-binding response regulator VraR
SAOUHSC_02099	1.4977	1.40E-28	5.17E-28	Histidine kinase
SAOUHSC_02314	–1.5182	4.87E-39	2.29E-38	Sensor protein KdpD
SAOUHSC_01313	–1.245	4.20E-07	5.34E-07	Histidine kinase
SAOUHSC_02262	–1.9379	1.40E-11	2.53E-11	Hypothetical protein
SAOUHSC_00184	–1.0885	0.0024819	0.001952	Response regulator receiver domain-containing protein
SAOUHSC_00183	–1.0596	5.73E-08	8.01E-08	Sugar phosphate antiporter
SAOUHSC_00233	–3.2508	1.79E-232	3.94E-231	Antiholin-like protein LrgB
SAOUHSC_00232	–3.6781	4.09E-107	4.33E-106	Murein hydrolase regulator LrgA
SAOUHSC_00714	–2.4109	7.455E-159	1.19E-157	Sensor histidine kinase SaeS
SAOUHSC_00715	–2.5865	1.4646E-88	1.34E-87	Response regulator
**Transcriptional regulator**				
SAOUHSC_01685	1.5865	9.95E-181	1.72E-179	Heat-inducible transcription repressor HrcA
SAOUHSC_00234	1.459	0.00068977	0.000593	Hypothetical protein
SAOUHSC_02098	1.8525	9.03E-37	4.06E-36	DNA-binding response regulator VraR
SAOUHSC_01574	2.7087	0.003039	0.002356	Helix-turn-helix domain-containing protein
SAOUHSC_02388	–1.2644	1.54E-25	5.19E-25	Hypothetical protein
SAOUHSC_02589	–1.249	8.64E-52	4.99E-51	Hypothetical protein
SAOUHSC_00070	–1.7513	2.77E-35	1.21E-34	Accessory regulator-like protein
SAOUHSC_01897	–1.7701	0.00011868	0.000115	Hypothetical protein
SAOUHSC_01314	–1.3492	1.16E-08	1.74E-08	Hypothetical protein
SAOUHSC_02532	–1.3201	6.17E-07	7.77E-07	Hypothetical protein
SAOUHSC_01045	–1.4138	1.45E-08	2.14E-08	Hypothetical protein
SAOUHSC_01879	–1.0061	1.55E-17	3.80E-17	Virulence factor regulator protein
SAOUHSC_00314	–1.4123	8.68E-27	3.02E-26	Hypothetical protein
SAOUHSC_01891	–1.5254	0.0042606	0.003217	Arsenate operon regulator
SAOUHSC_01655	–1.7145	1.57E-12	3.00E-12	Hypothetical protein
SAOUHSC_00291	–1.2486	5.11E-06	5.72E-06	PfkB family carbohydrate kinase
SAOUHSC_01997	–1.2286	4.00E-290	1.12E-288	Ferric uptake regulator-like protein
**Virulence**				
SAOUHSC_02708	–4.7004	6.027E-148	9.3E-147	Gamma-hemolysin h-gamma-II subunit
SAOUHSC_01110	–4.3545	1.8411E-32	7.49E-32	Fibrinogen-binding protein-like protein
SAOUHSC_02706	–2.9794	2.2882E-91	2.17E-90	Immunoglobulin G-binding protein Sbi
SAOUHSC_01121	–2.9556	1.1658E-25	3.97E-25	Alpha-hemolysin
SAOUHSC_02243	–5.8235	5.11E-136	6.70E-135	Hypothetical protein
SAOUHSC_01179	1.5781	4.96E-40	2.40E-39	Primosomal protein N
SAOUHSC_02241	–5.1997	1.86E-146	2.76E-145	Hypothetical protein
**Cell adhesion**				
SAOUHSC_00366	1.2455	5.24E-30	2.01E-29	NAD(P)H-flavin oxidoreductase
SAOUHSC_00812	1.5722	0	0	Clumping factor
SAOUHSC_02101	1.6223	2.34E-07	3.07E-07	Hypothetical protein
SAOUHSC_02047	1.217	0.0005159	0.0004584	Phage head morphogenesis protein
SAOUHSC_02182	2.0715	2.47E-10	4.12E-10	Tail length tape measure protein
SAOUHSC_00143	–2.9349	5.66E-103	5.83E-102	Hypothetical protein
SAOUHSC_00544	–2.7801	7.18E-20	2.00E-19	Fibrinogen-binding protein SdrC
SAOUHSC_00545	–1.1278	3.09E-07	4.01E-07	Fibrinogen-binding protein SdrD

*The log2 (fold-change) indicates the multiples of change of FA-JP21 gene compared with JP21, *p*-value is used to determine statistically significant indicators, *q* value is the corrected *p*-value, and description is based on NCBI annotations for gene description.*

### Subinhibitory Concentration of Fusidic Acid Affects the α-Toxin of *Staphylococcus aureus*

#### Influence of Subinhibitory Concentrations of Fusidic Acid on α-Toxin Production of *Staphylococcus aureus*

**FIGURE 3:**
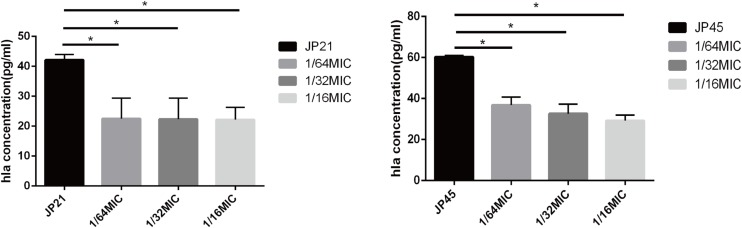
Effect of a subinhibitory concentration of fusidic acid on α-toxin (Hla) release was quantified by ELISA in *Staphylococcus aureus* treated with or without fusidic acid. *p < 0.05.

By transcriptome sequencing, we found that the expression of *hla* encoding α-toxin associated with *S. aureus* virulence decreased substantially, and then we used an ELISA kit to detect α-toxin specific production. After being treated with fusidic acid (1/32 × MIC), the concentrations of α-toxin decreased from 36.36 to 26.24 pg/ml for JP21 and from 60.27 to 29.23 pg/ml for JP45 (Figure 3).

#### The Effect of Subinhibitory Concentrations of Fusidic Acid on α-Toxin Activity of *Staphylococcus aureus*

**FIGURE 4:**
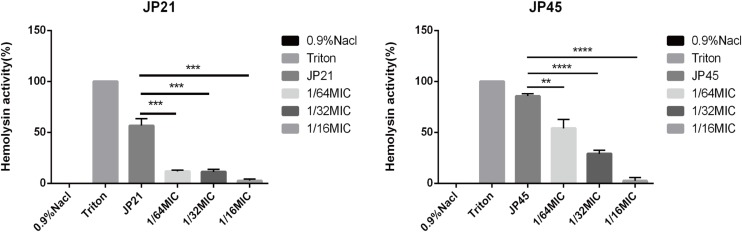
Effects of different subinhibitory concentrations of fusidic acid on hemolytic activity of *Staphylococcus aureus*. 0.9% NaCl and Triton used as negative control and positive control, respectively. There were significant differences between the treated group (treated with fusidic acid) and the untreated group. **p < 0.01, ***p < 0.001, ****p < 0.0001.

Then, we investigated the effect of subinhibitory concentrations of fusidic acid on α-toxin activity. We evaluated the effect of fusidic acid on the α-toxin activity of *S. aureus* supernatants by comparing the percentage of hemolysis with that of the group untreated with fusidic acid. The α-toxin activity of the fusidic acid treated group was obviously lower than that of the untreated group (Figure 4). For JP21, the hemolysis rate of the untreated group was 60.67%, and after being treated with fusidic acid, its rate decreased to 3.52–12.74%. For JP45, the hemolysis rate of the untreated group was 86.95%, and after being treated with fusidic acid, its rate decreased to 4.41–59.05%. And the decrease in hemolytic activity is dependent on the concentration of the drug. Moreover, the solvent has no effect on the hemolytic activity (Supplementary Figure S2).

### Subinhibitory Concentrations of Fusidic Acid Influence the *hla* Expression of *Staphylococcus aureus*

**FIGURE 5:**
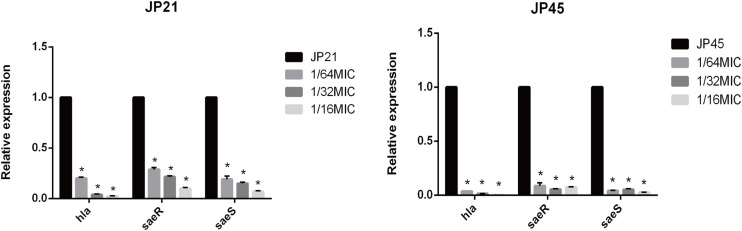
Relative expression of *hla* and *saeR/S* in *Staphylococcus aureus* strains treated with various subinhibitory concentrations of fusidic acid [1/64, 1/32, and 1/16 × minimum inhibitory concentration (MIC)]. Values are means ± SDs (based on three repeated assays). There were significant differences with the control group (grown without fusidic acid) for each strain (*p < 0.05).

By transcriptome sequencing, we found a significant decrease in *hla* expression, so we first verified the expression of *hla* from the mRNA level. We found that the relative expression of *hla* in *S. aureus* clinical isolates treated with different subinhibitory concentrations of fusidic acid was significantly decreased (Figure 5). After incubation with the subinhibitory concentrations of fusidic acid (1/64, 1/32, and 1/16 × MIC), the relative expression of *hla* decreased by 4.77-fold to 51.17-fold in JP21 and 35.52-fold to 309.66-fold in JP45. In order to investigate the mechanism of expression of α-toxin inhibited by the subinhibitory concentrations of fusidic acid, we performed RT-PCR to investigate the expression of *saeRS* associated with the expression of *hla.* We found that the relative expression of *saeRS* in *S. aureus* isolates treated with different subinhibitory concentrations of fusidic acid was significantly decreased (Figure 5), conforming to the results of transcriptome sequencing. After incubation with the subinhibitory concentrations of fusidic acid (1/64, 1/32, and 1/16 × MIC), the expression of *saeR* decreased by 3.29-fold to 11.18-fold in JP21 and 8.51-fold to 21.89-fold in JP45, respectively. The expression of *saeS* matched that of *saeR*.

### Subinhibitory Concentration of Fusidic Acid Affects the Formation of *Staphylococcus aureus* Biofilm

#### Fusidic Acid Affects the Formation of *Staphylococcus aureus* Biofilm

**FIGURE 6:**
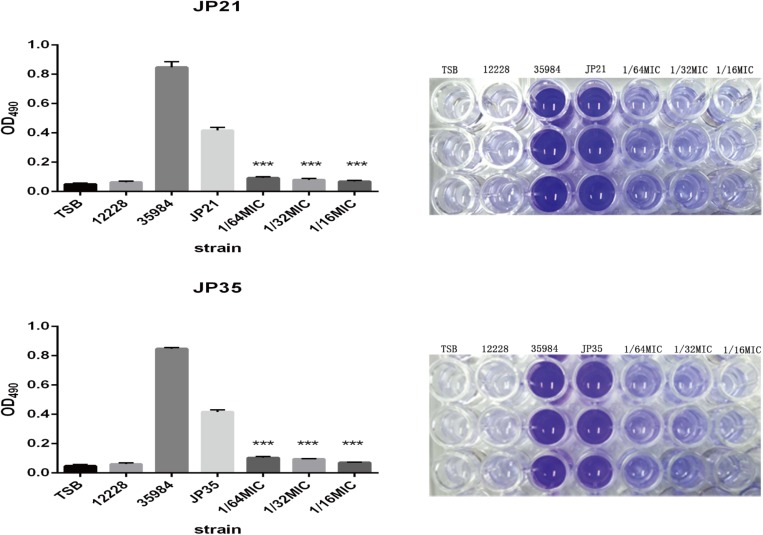
Effects of different subinhibitory concentrations of fusidic acid on biofilm formation of *Staphylococcus aureus*. On the right is the picture after dissolution with glacial acetic acid, and on the left is the optical density (OD) value measured at 490 nm. *Staphylococcus* spp. 12228 and 35884 served as the negative control and positive control, respectively. There were significant differences with the control group (grown without fusidic acid) for each strain (***p < 0.0001). Each test was performed independently in triplicate.

**FIGURE 7:**
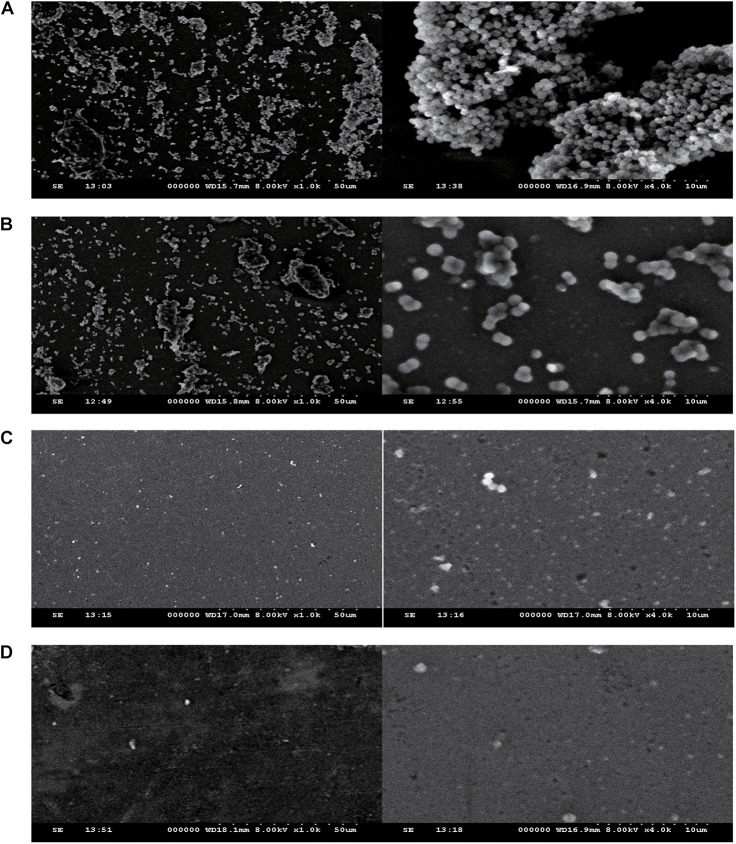
The effect of different subinhibitory concentrations of fusidic acid(1/64, 1/32, and 1/16 × minimum inhibitory concentration (MIC) on the biofilm of *S. aureus* (JP21) was detected by scanning electron microscopy. The image on the left side was magnified 1,000 times, and the image on the right side was magnified 4,000 times. **(A)** JP21 without fusidic acid. **(B)** JP21 with 1/64 × MIC fusidic acid. **(C)** JP21 with 1/32 × MIC fusidic acid. **(D)** JP21 with 1/16 × MIC fusidic acid.

The effect of fusidic acid on *S. aureus* biofilm formation was assessed by biofilm semi-quantitative assay and scanning electron microscopy in comparison with that of the untreated group. Fusidic acid treatment depressed the formation of *S. aureus* biofilm when the concentrations were up to 1/64 × MIC (Supplementary Figure S3). The formation of *S. aureus* biofilm of the experimental group without drug treatment was significantly stronger than that of groups treated with different concentrations of fusidic acid (from 1/64 to 1/16 × MIC) in a concentration-dependent manner (Figures 6, 7). The biofilm formed by the bacteria added with fusidic acid was significantly thinner than the unmedicated one. And the solvent has no effect on the formation of biofilm (Supplementary Figure S4).

#### Fusidic Acid Affects the Autolysis Ability of *Staphylococcus aureus*

**FIGURE 8:**
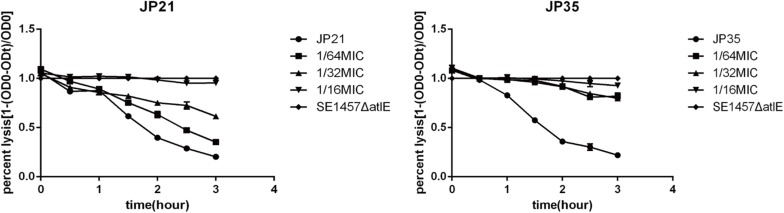
The effect of different subinhibitory concentrations of fusidic acid [1/64, 1/32, and 1/16 × minimum inhibitory concentration (MIC)] on the autolysis of *Staphylococcus aureus*. *Staphylococcus* 1457 ΔatlE as a negative control. Each test was performed independently in triplicate.

Related literature (Rice et al., 2007) indicates that the autolysis ability of bacteria is related to the formation of biofilm, and the stronger the autolysis ability, the stronger the biofilm formation. In our study, the autolysis ability of *S. aureus* with added fusidic acid was significantly decreased compared with that of the untreated *S. aureus* (Figure 8).

### Subinhibitory Concentration of Fusidic Acid Affects the Aggregation of *Staphylococcus aureus*

**FIGURE 9:**
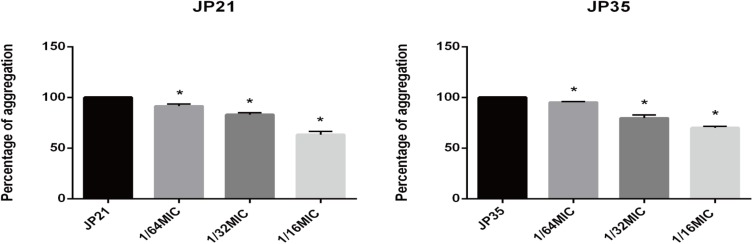
The effect of different subinhibitory concentrations of fusidic acid [1/64, 1/32, and 1/16 × minimum inhibitory concentration (MIC)] on the cell aggregation of *Staphylococcus aureus*. Significantly lower than the value for the untreated control (*p* < 0.05). Each test was performed independently in triplicate.

The cell aggregation plays an indispensable role in the formation of biofilms. We can see a significant decrease in cell aggregation of *S. aureus* after treatment with fusidic acid (Figure 9). After the addition of 1/64 × MIC fusidic acid, the relative cell aggregation of *S. aureus* decreased to 91.42–95.2% and 79.92–83.05% when 1/32 × MIC fusidic acid was added and 63.32–70.11% when 1/16 × MIC fusidic acid was added.

### Subinhibitory Concentration of Fusidic Acid Affects the Production of Polysaccharide Intercellular Adhesin of *Staphylococcus aureus*

**FIGURE 10:**
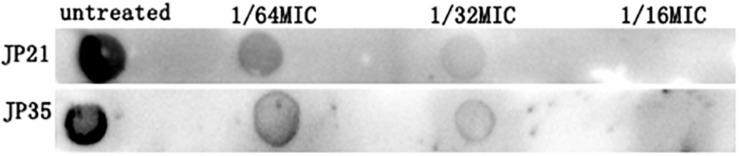
The effect of subinhibitory concentrations of fusidic acid [1/64, 1/32, and 1/16 × minimum inhibitory concentration (MIC)] on intercellular polysaccharide adhesin of *Staphylococcus aureus*.

The extracellular PIA is identified as the main component of the staphylococcal biofilm (Paharik and Horswill, 2016). Our experimental results showed that the intercellular polysaccharide adhesin produced by *S. aureus* added with fusidic acid was significantly reduced (Figure 10).

### Subinhibitory Concentrations of Fusidic Acid Down-Regulated the Expression of *cidA*, *icaA*, *sarA*, and *spa* Genes of *Staphylococcus aureus*

**FIGURE 11:**
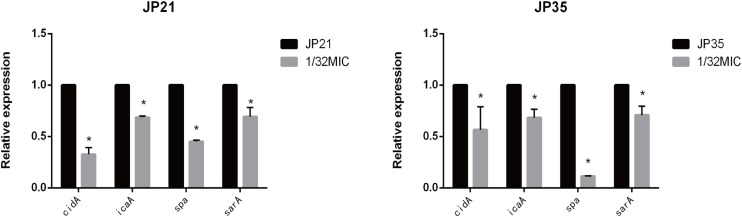
Relative expression of genes associated with *Staphylococcus aureus* biofilm cultured in 1/32× minimum inhibitory concentration (MIC) fusidic acid. Values are means ± SDs (based on three repeated assays). There were significant differences with the control group (grown without fusidic acid) for each strain (*p < 0.05).

In order to gain a deeper understanding of the subinhibitory concentrations of fusidic acid and how to inhibit the biofilm of *S. aureus*, we performed RT-PCR. We extracted *S. aureus* wild (not cultured with fusidic acid) and treated (with 1/32 × MIC fusidic acid) RNA, and then we performed RT-PCR experiments and found that the expression of *cidA*, *icaA*, *spa*, and *sarA* gene treatments has varying degrees of decline (Figure 11).

### Subinhibitory Concentrations of Fusidic Acid Reduces the Pathogenicity of *Staphylococcus aureus*

**FIGURE 12:**
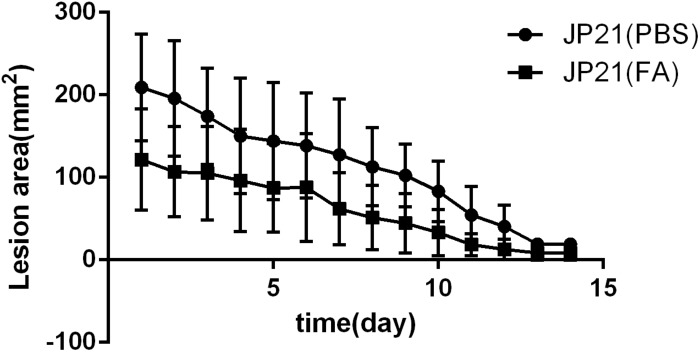
Experimental results of a mouse skin abscess model. JP21 [phosphate-buffered saline (PBS)] was the untreated group and JP21 (FA) was the treatment group.

The mouse skin abscess model experiments were divided into two groups: one group was the untreated group, and the other group was the treatment group (treated with fusidic acid daily). The average lesion area of the untreated group was 112.187 mm^2^, and the average lesion area of the treatment group was 60.241 mm^2^. The lesion area of the treatment group was significantly smaller than that of the untreated group throughout the trial (Figure 12).

## Discussion

*Staphylococcus aureus* has always been an important pathogen of community and nosocomial infections. *S. aureus* can causes multiple infections in humans, including relatively mild infections such as skin and soft tissue infections, as well as severe life-threatening invasive diseases such as bacteremia, pneumonia, and endocarditis. Owing to the emergence of multidrug-resistant *S. aureus* and an increasing trend, many research efforts have begun to shift the focus on how to reduce the virulence of *S. aureus* instead of the traditional through removing pathogens. In recent years, many studies have demonstrated that subinhibitory concentrations of antibiotics have an effect on *S. aureus* virulence factors (Otto et al., 2013) and biofilm formation (Majidpour et al., 2017). But it is more than subinhibitory antibiotics enhancing the virulence of *S. aureus* (Hodille et al., 2017).

Fusidic acid is often used to treat a variety of infections caused by Gram-positive bacteria and is particularly effective against infections caused by staphylococci. In recent years, it has been reported in the literature that fusidic acid has an inhibitory effect on the biofilm formation of *S. aureus* (Siala et al., 2018), but the mechanism is not clear. Inspired by the subinhibitory concentrations of mupirocin and resveratrol, which can down-regulate the virulence of *S. aureus* in our previous study (Duan et al., 2018; Jin et al., 2018), we attempted to study the effect of subinhibitory concentrations of fusidic acid on the virulence of *S. aureus*.

We selected three different sequence-type (ST) clinical *S. aureus* strains that were resistant to fusidic acid to study the effect of subinhibitory concentrations of fusidic acid on the virulence of *S. aureus*. We found that the high concentrations of the fusidic acid inhibited the growth of *S. aureus* (Supplementary Figure S1), so we chose three subinhibitory concentrations that have no effect on the growth of *S. aureus* in our study, eliminating the virulence caused by the reduction of bacteria. Through transcriptome sequencing, we found that the expression of many genes in *S. aureus* treated with fusidic acid was down-regulated; it shows that fusidic acid can affect the expression of *S. aureus* gene, including genes associated with virulence factors and biofilm, such as *hla*, *icaA*, and *spa*. It has been reported that molecular compounds or antibiotics can reduce the virulence of *S. aureus* by reducing the expression of virulence-associated genes (Kebaier et al., 2012; Otto et al., 2013). α-Toxin (encoded by *hla*) plays an important role in *S. aureus* skin and soft tissue infections (Shallcross et al., 2010; Nygaard et al., 2012), and it can increase the abscess and necrosis of the infected parts. We suggested that the subinhibitory concentrations of fusidic acid may reduce the virulence of *S. aureus* by down-regulating the expression of *hla* and biofilm formation.

For the part of the α-toxin, the ELISA kit quantitative α-toxin test and α-toxin activity test confirmed our hypothesis that the subinhibitory concentrations of fusidic acid can significantly inhibit the amount of α-toxin production and inhibit the α-toxin activity of the experimental strain. The production of α-toxin of *S. aureus* is regulated by a variety of regulatory systems, of which *saeRS* is considered to be the most important regulatory system, because the sae locus is important for *hla* transcription (Liu et al., 2016). By RT-PCR, we found that the subinhibitory concentrations of fusidic acid down-regulated the expression of *saeRS* at the mRNA level. This is consistent with previous research in our laboratory (Duan et al., 2018; Jin et al., 2018) in that *saeRS* can down-regulate the production of α-toxin. For the part of the biofilm, it was found by the crystal violet staining method that the *S. aureus* biofilm with fusidic acid was down-regulated, which is consistent with the previous study (Siala et al., 2018). After that, it was confirmed by scanning electron microscopy that the expression of biofilm was indeed down-regulated. The formation of *S. aureus* biofilm can be roughly divided into three steps: first, initial attachment, followed by biofilm accumulation caused by intercellular aggregation (maturation), and finally bacterial cell separation caused by the direct action of bacterial products (dispersion) (Periasamy et al., 2012). We did cell aggregation experiments and found that the aggregation of *S. aureus* was significantly reduced after the addition of fusidic acid. Different bacteria have different mechanisms of biofilm formation. Among *S. aureus*, methicillin-sensitive strains mostly require the PIA, whereas methicillin-resistant strains are most often formed independent of PIA, but in a glucose-dependent manner (DeFrancesco et al., 2017). In *S. aureus*, PIA plays an important role in the adhesion and aggregation of biofilm formation and plays a key role in the adhesion between bacteria (Gotz, 2002), which is mainly synthesized by the expression of *icaA* gene. In this study, we examined PIA by enzyme-linked spot immunoassay and found that the PIA production of *S. aureus* with fusidic acid was significantly reduced. Moreover, *sarA* can up-regulate biofilm formation by affecting *ica* transcription and producing PIA (Valle et al., 2003; Beenken et al., 2004). Our experimental results indicate that the subinhibitory concentrations of fusidic acid can reduce the expression of *icaA* and *sarA* at the mRNA level. Also, it has been reported in the literature that autolysis promotes the formation of *S. aureus* biofilm (Rice et al., 2007). The *cidABC* and *lrgAB* of *S. aureus* operons regulate cell lysis in an opposing manner: *lrgAB* has an inhibitory effect on murein hydrolase activity (Groicher et al., 2000), whereas *cidA* has a positive effect on murein hydrolase activity (Rice et al., 2003; Rice et al., 2005). Our study found that fusidic acid can reduced the expression of *cidA* and autolysis of *S. aureus*. Staphylococcal protein A (SPA) is expressed by gene *spa*, which is an important virulence factor of *S. aureus* and plays an important role in the adhesion and aggregation of bacteria during biofilm formation (Ythier et al., 2012). We found that fusidic acid also reduced the expression of *spa*. Therefore, we speculate that fusidic acid reduces the formation of biofilm by reducing the adhesion, aggregation, and autolysis of *S. aureus*. Fusidic acid down-regulates the expression of *icaA* by down-regulating *sarA*, which reduces the production of PIA and also down-regulates the expression of *spa*, thereby reducing the adhesion and aggregation of *S. aureus*. The autolysis of *S. aureus* was down-regulated by down-regulating the expression of *cidA*.

In addition, our mouse skin abscess model test results show that fusidic acid can reduce the damage area of the skin. It shows that fusidic acid can effectively reduce the pathogenicity of *S. aureus*.

In summary, our study found that the subinhibitory concentrations of fusidic acid can reduce the virulence of *S. aureus* by inhibiting the expression of α-toxin and biofilm formation. We speculate that the possible reason is to reduce the expression of α-toxin by down-regulating *saeRS* and to reduce biofilm formation by inhibiting cell adhesion, aggregation, and autolysis. This study not only provides a new method for the treatment of *S. aureus* infection but also broadens the clinical application of fusidic acid. This has great enlightenment for the new use of common drugs.

## Data Availability Statement

All datasets generated for this study are included in the article/Supplementary Material.

## Ethics Statement

The animal study was reviewed and approved by the Ethics Committee of Wenzhou Medical University.

## Author Contributions

LL, XS, JY, and XC designed the work and analyzed and interpreted data for the work. QZ and LL drafted the work and revised it critically for important intellectual content. FY provided approval for publication of the content, and agreed to be accountable for all aspects of the work in ensuring that questions related to the accuracy or integrity of any part of the work are appropriately investigated and resolved. YG participated in the experimental design and data analysis. All authors read and approved the final manuscript.

## Conflict of Interest

The authors declare that the research was conducted in the absence of any commercial or financial relationships that could be construed as a potential conflict of interest.
