# Supplementary material for: Subinhibitory Concentrations of Fusidic Acid May Reduce the Virulence of S. aureus by Down-Regulating sarA and saeRS to Reduce Biofilm Formation and α-Toxin Expression
Source: Front Microbiol. 2020 Feb 14;11:25. doi: 10.3389/fmicb.2020.00025 (PMC7033611; doi:10.3389/fmicb.2020.00025)
Supplement: Supplementary file 1 [file Data_Sheet_1.pdf]

Supplementary Material

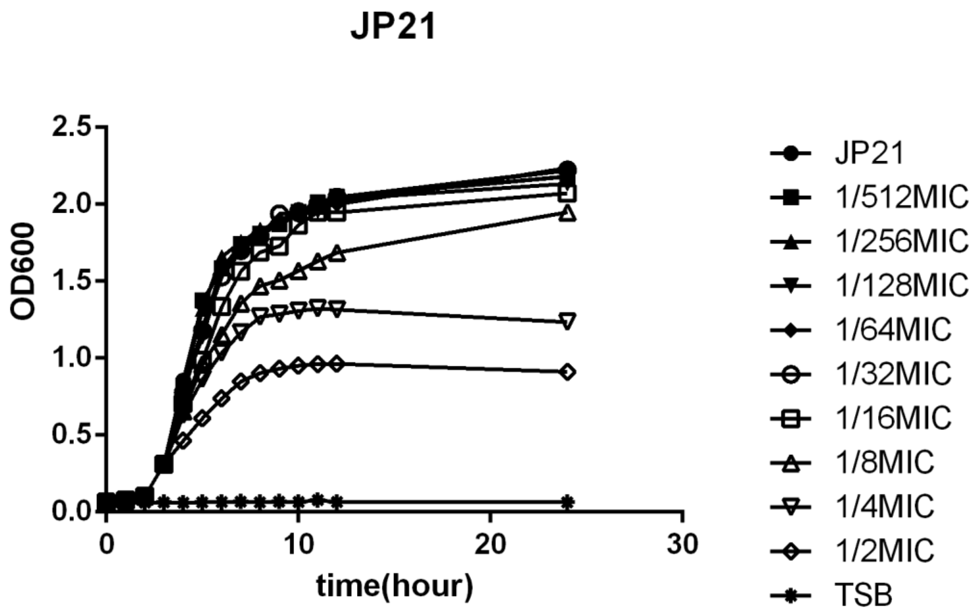

Figure S1. Growth curve of *S.aureus* JP21 at different sub-inhibitory concentrations of fusidic acid.

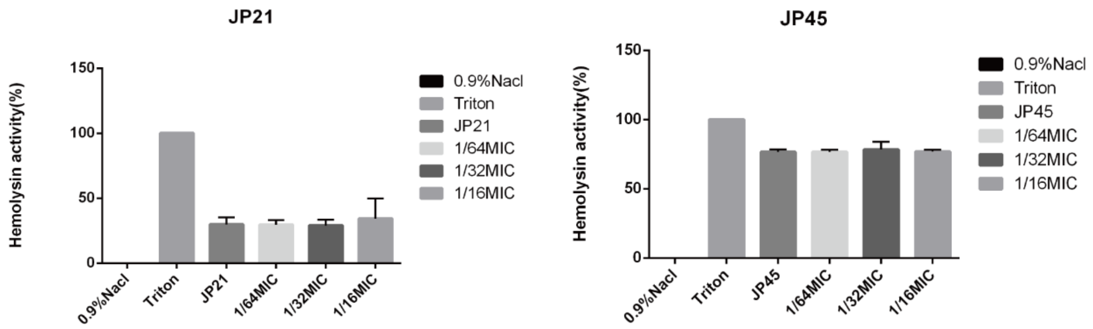

Figure S2. The same amount of solvent (95% ethanol) as the corresponding concentration of fusidic acid had no effect onthe hemolytic activity of *S. aureus*. 0.9% Nacl and Triton used as negative control and positive control, respectively. Each test was performed independently in triplicate.

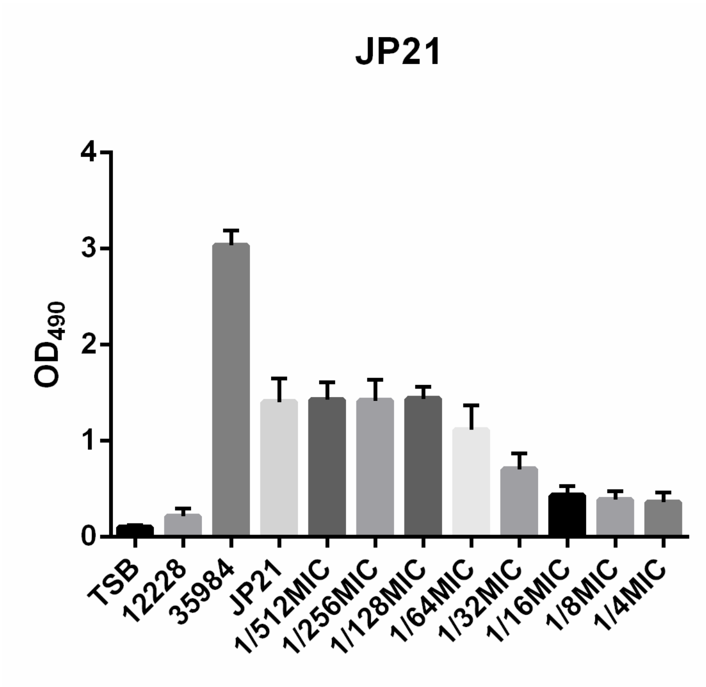

Figure S3. The effect of different sub-inhibitory concentrations of fusidic acid on the formation of JP21 biofilm.

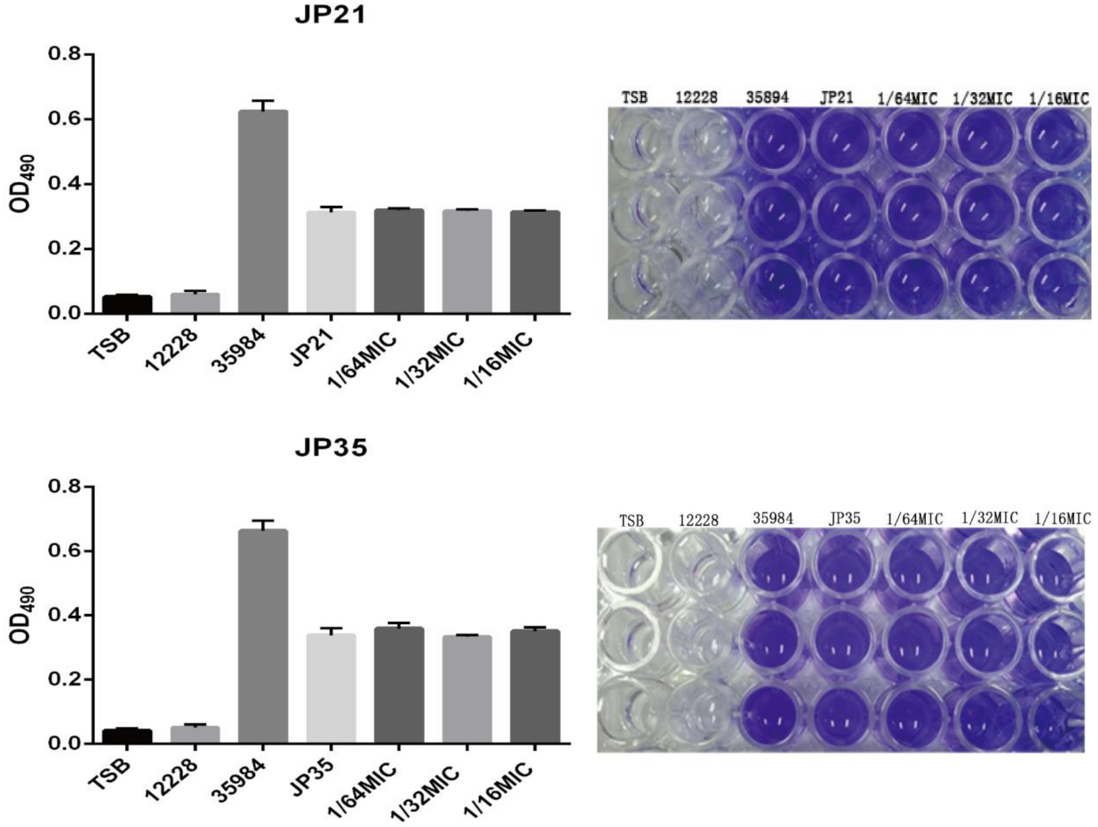

Figure S4. The same amount of solvent (95% ethanol) as the corresponding concentration of fusidic acid had no effect onthe formation of *S. aureus* biofilm. On the right is the picture after dissolution with glacial acetic acid, and on the left is the OD value measured at 490 nm. *Staphylococcus sp. 12228*, *35884* as negative control and positive control, respectively. There was no difference in the control group (growth without fusidic acid) for each strain. Each test was performed independently in triplicate.

Table S1 Primers used for real-time RT-PCR

Primer name	Sequence (5 mers u
gyrb-RT-F	ACATTACAGCAGCGTATTAG
gyrb-RT-R	CTCATAGTGATAGGAGTCTTCT
icaA-RT-F	GTTGGTATCCGACAGTATA
icaA-RT-R	CACCTTTCTTACGTTTTAATG
cidA-RT-F	TAGCGTAATTTCGGAAGC
cidA-RT-R	GGCAGTATTGTTGGTCTA
sarA-RT-F	AAACCCTGAATTTGAATG
sarA-RT-R	GATATTACATCTGCTCCT
spa-RT-F	AGGCTTGTTGTTGTCTTC
spa-RT-R	CCTTCAGTGAGCAAAGAA
hla-RT-F	GTCATTTCTTCTTTTTCCCAATCG
hla-RT-R	CACGTATAGTCAGCTCAGTAACA
saeR-RT-F	GTCGTAACCATTAACTTCTG
saeR-RT-R	ATCGTGGATGATGAACAA
saeS-RT-F	TGTATTTAAAGTGATAATATGAG
saeS-RT-R	CTTAGCCCATGATTTAAAACA
